# Topical *Hypericum perforatum* Improves Tissue Regeneration in Full-Thickness Excisional Wounds in Diabetic Rat Model

**DOI:** 10.1155/2015/245328

**Published:** 2015-08-31

**Authors:** Soheila Yadollah-Damavandi, Mehdi Chavoshi-Nejad, Ehsan Jangholi, Noushin Nekouyian, Sahar Hosseini, Amin Seifaee, Shima Rafiee, Hossein Karimi, Soheil Ashkani-Esfahani, Yekta Parsa, Maryam Mohsenikia

**Affiliations:** ^1^Young Researchers and Elite Club, Islamic Azad University, Tehran Medical Sciences Branch, Tehran, Iran; ^2^Student Research Committee, Shahid Beheshti University of Medical Sciences, Tehran, Iran; ^3^Student Research Committee, Shiraz University of Medical Sciences, Shiraz, Iran; ^4^Dezful University of Medical Sciences, Dezful, Iran

## Abstract

Delayed wound healing process is one of the most important concerns in diabetes. Healing of wounds has four phases, namely, hemostasis, inflammation, proliferation, and remodeling. For a successful repair, all four factors must occur properly. Hence, we aimed to evaluate the healing effects of* Hypericum perforatum* (HP) on full-thickness diabetic skin wounds by using stereological methods. Forty-eight female diabetic rats were randomly divided into four groups (*n* = 12): gel base treated group, HP 5% gel treated group, HP 10% gel treated group, and the control group which received no treatment. A circular 1 cm^2^ full-thickness wound was created on the animal's neck and wound area was measured every three days. After sacrificing the animals, skin samples were fixed and prepared for stereological evaluations. Based on the results, HP treated group showed faster wound closure rate in comparison with control and vehicle groups (*P* < 0.05). In addition, numerical density of fibroblasts, volume density of collagen bundles, and mean diameter and volume densities of the vessels in HP group were significantly higher than control and vehicle groups. The results of this study showed that HP has the ability to improve tissue regeneration by enhancing fibroblast proliferation, collagen bundle synthesis, and revascularization.

## 1. Introduction

It is reported that there are more than 347 million people with diabetes all over the world [1]. One of the most important concerns of diabetic patients is delayed wound healing which can lead to serious complications such as limb amputations, organ dysfunctions, cosmetic impairments, infections, sepsis, and even death [2]. Actually wound healing as a dynamic, multicellular, and normal biological process in the human body has four phases: hemostasis, inflammation, proliferation, and remodeling; for a successful repair all four factors must occur properly [3]. Diabetic patients suffer from impaired vascularization, impaired tissue regeneration, collagen synthesis, and immune system [4–6]. Thus, they may have problem in the process of wound healing which may be assisted by additional treatments, either medical or surgical treatments. Moreover, anti-inflammatory and antioxidant responses are considerably compromised in diabetes [7, 8].

Medicinal plants have recently received much attention for their various medicinal properties.* Hypericum perforatum* (HP), a member of the genus* Hypericum* family, is found in Europe, West Asia, North Africa, Madeira, and the Azores [9]. The extract of HP contains naphthodianthrones, phloroglucinols, flavonoids, bioflavonoids, and phenylpropanoids which promote the wound healing process and have antifungal, anti-inflammatory, antimycobacterial, and antiviral activities [9–12]. The plant has a wide range of medicinal applications such as skin wounds, eczema, burns, diseases of the alimentary tract, and psychological disorders [11]. In this research, we aimed to investigate the effects of HP on diabetic wound healing in rat models by using stereological parameters.

## 2. Materials and Methods

### 2.1. Preparation of Plant Extract and Vehicle Gel

All organs of the HP plant were used as plant material for processing the extract, dried at room temperature for 4–7 days. The dried material was ground into powder and subsequently extracted with a mixture of water : ethanol (1 : 1, v/v) for 72 hours. The obtained material was then filtered and the filtrate was evaporated to obtain a dark hydroalcoholic extract (yield: 24.25%). In order to facilitate the application of the agent, we provided 5% and 10% HP gel by dissolving 5 cc (HP 5) and 10 cc (HP 10) of the extract each in 2 cc distilled water and then transferred the solution into 2% carboxymethylcellulose (CMC) (2 g CMC dissolved in 98 cc distilled water) [13]. The dosages were selected according to a previous pilot study which was conducted for finding the lowest effective doses of the extract. The gel base was also supplied by creating 2% CMC gel without the HP component.

### 2.2. Animals and Excision of Wound Model

In this experimental study, 48 female Wistar rats (200 ± 20 g; 2-3 months old) kept in standard cages with food and water* ad libitum* were divided into four groups randomly (*n* = 12): gel base (vehicle) treated group, HP 5% gel treated group, HP 10% gel treated group, and the control group which received no treatment.

Diabetes was induced by administration of 50 mg/kg streptozotocin (Sigma Aldrich, Germany) intraperitoneally to the rats. The blood sugar was estimated by using a glucometer device (Bionime) and the ones with blood sugar higher than 250 were considered as diabetic. On day 0, rats were wounded (a 1 cm^2^ circular full-thickness skin wound) under general anesthesia induced by ether inhalation on the posterior surface of their necks. The topical administrations were done in a standard manner just after the wounding and were repeated every 24 h until the last day of the study (day 15). The last day of the study was assigned as the day in which at least one of the wounds in any group was closed. The animals were sacrificed with a high dose of ether on day 15. Full-thickness skin samples (1 × 1 cm) were provided from the wound site and were fixed in buffered formaldehyde (pH = 7.2) for further procedures.

The study protocol was approved by the Medical Ethics Committee of Shiraz University of Medical Sciences and the animal care was in accordance with the related guidelines.

### 2.3. Preparation of the Samples and Stereological Study

Stereological study was performed according to previously used methods in this field by Ashkani-Esfahani et al. [13]. To determine the wound closure rate by using the application of stereology, images were captured from the wound surfaces every three days with a digital camera. To calibrate the magnification, a standard ruler was set at the level of the wound in each photograph, and the wound areas at the first and last day of experimental study were estimated by using a stereology software composed of a point grid and by using the following formula: Area = ∑*P* × *a*/*p*, where ∑*P* was the total points laid on the wound area and *a*/*p*, the area surrounded by every four crosses, was considered as the area per point (mm^2^) [13]. Thereafter, the wound closure rate was calculated as follows: wound closure rate (%) = ((area at the 1st visit − area at each visit)/area at visit 1) × 100.

Nine pieces of the skin samples, each about 1 mm^2^, were cut and prepared in a systematic random sampling manner for stereological analysis. The pieces of each sample were embedded in a paraffin block. Isotropic uniform random (IUR) sections of the blocks with 5 and 15 *μ*m thickness were created and stained with H&E and Heidenhain's Azan-trichrome stain [13]. Microscopic analyses of the dermis were performed by using a video-microscopy system made up of a microscope linked to a camera (Alpha-200; Sony; Japan) and a flat monitor. The volume densities of the collagen bundles, vessels, and hair follicles (*Vv*: fraction of the unit volume of the dermis which is occupied by the collagen bundles, vessels, or hair follicles) were estimated by using the stereological point counting method and the following formula: *Vv*_(collagen  or  vessel  or  hair  follicle/dermis)_ = *P*_(collagen  or  vessel  or  hair  follicle)_/*P*_(dermis)_, where *P*_(collagen  or  vessel  or  hair  follicle)_ was the number of points hitting the profiles of the collagen bundles, vessels, or hair follicles and *P*_(dermis)_ was the number of points hitting the reference visible field (dermis).

The numerical density (*Nv*: number of cells per unit volume of the dermis) of the fibroblasts was estimated by employing the 15 *μ*m slides, the “optical dissector” method [13], and the following formula: *Nv* = ∑*Q*/∑*A* × *h*, where “∑*Q*” is the number of nuclei coming into focus in the dissector height, “Σ*A*” is the total area of the counting frame in all microscopic fields, and “*h*” is the height of dissector within which the counting is done. The upper and the lower 5 *μ*m were considered as “area of safety.”

### 2.4. Statistical Analysis of the Data

Final results were collected and reported as mean and standard deviation (mean ± SD). Besides, the statistical comparisons between the groups were done by the SPSS statistical software (v.14.0). Considering the nonparametric data in this study, Kruskal-Wallis test and Mann-Whitney *U* test were used in order to analyze the data and compare the groups [13]. Moreover, *P* = 0.05 was considered as statistically significant.

## 3. Results

### 3.1. Area of the Wounds

**Figure 1:**
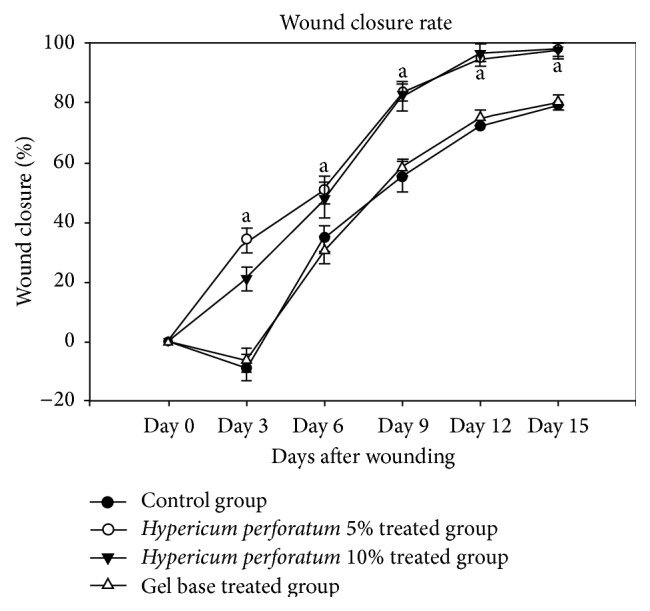
The effect of* Hypericum perforatum* (HP) on the wound closure rate. The groups consisted of the control, gel base treated, and HP 5% and 10% concentration gels treated rats. Each point represents mean ± SD of the wounds. The letter “a” shows significant difference in the HP 5% and 10% treated rats compared to the control group and the base group (*P* < 0.05).

The mean initial area of the wounds was 103.53 ± 7.11 mm^2^ (range 93.13–110.54 mm^2^) with no significant difference among the four groups. However, the rate of wound closure in the HP 5% (8.61%/day) and HP 10% (6.80%/day) treated groups was significantly higher (*P* < 0.05) in comparison to the base (4.92%/day) and the control groups (4.42%/day) (Figure 1). According to Figure 1, the wound areas increase in control and gel base groups up to day 3 and then decrease with a slope almost similar to the HP treated groups till the end of the study.

### 3.2. Fibroblast Population

The populations of the fibroblasts in the dermis of the HP treated groups were considerably higher than those of control and gel base groups. The populations of the fibroblasts in the HP 5% and HP 10% treated groups were 59.61% (*P* = 0.023) and 51.07% (*P* = 0.034) higher than the controls, respectively, and 96.49% (*P* = 0.009) and 85.98% (*P* = 0.013) higher than the gel base group, respectively (Table 1).

### 3.3. Volume Density of the Collagen Bundles and Hair Follicles

The volume densities of the collagen bundles were considerably higher by 54.21% (*P* = 0.028) and 56.75% (*P* = 0.026) in HP 5% and HP 10% treated groups compared to that of the control group (Table 1). In contrast with the gel base group, volume densities of the collagen bundles were significantly higher by 51.97% and 54.46%, respectively (*P* = 0.04). The volume densities of the hair follicles in the HP 5% and 10% treated groups showed insignificant differences compared to the gel base and the control groups.

### 3.4. Volume Density, Length Density, and Diameter of the Vessels

The length densities of the vessels in the HP 5% and 10% treated groups were significantly higher compared to the base group (*P* = 0.007 and *P* = 0.005, resp.) and the control group (*P* = 0.019 and *P* = 0.011, resp.). As Table 1 shows, there are also noticeable differences between the HP treated groups and the control and gel base groups regarding the mean diameter of the vessels (*P* < 0.05); however, no considerable differences were seen among the groups regarding the volume densities of the vessels.

## 4. Discussion

In this study, the effects of HP have been evaluated on the wound healing process in diabetic rats regarding stereological parameters including fibroblast population, collagen bundle synthesis, and revascularization. Our data showed that HP attenuates some of these parameters so that it results in hastened tissue regeneration.

There are not many studies published on the properties of HP which can be involved in the healing process by the way. According to present literature some influences of HP such as anti-inflammatory, antioxidant, antimicrobial, and antiviral activities are already reported as well as fibroblast proliferation inducing effect [9–12]. In previous in vitro and in vivo studies, impacts of HP have been demonstrated on the wound healing process. It is shown that the extract of this plant has tannins, hyperin, hypericin, hyperforin, amentoflavone, flavonoids, and xanthones [13]. It is also demonstrated that amentoflavone and hypericin have anti-inflammatory activities and also hyperforin has antibacterial potential [14]. Öztürk et al. studied the wound healing activity of HP extract on chicken embryonic fibroblasts. Based on their results, fibroblast percentage, collagen synthesis stimulation, and epithelial cell proliferation were increased in the presence of flavonoids and xanthones. In addition, flavonoids are known to reduce lipid peroxidation by preventing or slowing the onset of cell necrosis and improving vascularity [15]. Roa et al. have reported the effectiveness of oral HP alcoholic extract in wound healing of different animal models [16]. Lavagna et al. conducted a clinical study to investigate the effects of HP oily extract on cesarean wounds. Their results showed that this extract increases epithelial reconstruction and decreases perimeter surface areas which lead to healing promotion [17]. Also in another study, Samadi and his colleagues demonstrated that HP can facilitate incisional wound healing and reduce hypertrophic scar formation in women after caesarian operation [18].

Our results exhibited that topical administration of the medicinal herb, HP, enhances the tissue regeneration and wound healing process according to stereological parameters which makes this herb able to be introduced as an alternative treatment for skin damage. However, we still suggest further researches, especially clinical trials, in order to compare this agent with other herbal and chemical medicines and to figure out any possible side effects before any prescriptions.

## 5. Conclusion

The results of this study showed that HP has the ability to improve tissue regeneration by enhancing fibroblast proliferation, collagen bundle synthesis, and revascularization in diabetic skin injuries based on stereological analysis.

## Figures and Tables

**Table 1:** Mean + SD of the numerical density of the fibroblasts (×10^3^ per mm^3^), volume densities of the collagen bundles (%) and vessels (%), length density (mm/mm^3^), and the mean diameter (*μ*m) of the vessels in the dermis of the wounded rats treated with *Hypericum perforatum* 5% and 10% gels (HP5% and HP10%), those treated with gel base, and the untreated group (control).

Groups	Fibroblasts	Collagen	Hair	Vessels		
	Numerical density	% Volume density	% Volume density	% Volume density	Length density	Mean diameter
Control	284.5 (32.6)	47.4% (4.9%)	2.6% (1.2%)	3.3% (2.75%)	17.2 (8.1)	11.7 (3.1)
HP5%	454.1 (117.61)^*∗*^	73.1% (9.2%)^*∗*^	2.8% (1.6%)	3.1% (1.4%)	29.3 (6.1)^*∗*^	17.1 (5.5)^*∗*^
HP10%	429.8 (37.6)^*∗*^	74.3% (1.9%)^*∗*^	2.9% (1.2%)	5.1% (2.2%)	32.9 (16.6)^*∗*^	26.6 (9.4)^*∗*^
Gel base	231.1 (11.9)	48.1% (4.7%)	2.4% (1.3%)	4.1% (0.7%)	14.1 (1.4)	11.2 (1.8)

^*∗*^ *P* < 0.05, HP 5% and HP 10% treated groups versus control group and gel base group.
